# Effect of Platelet-Rich Plasma Addition on the Chemical Properties and Biological Activity of Calcium Sulfate Hemihydrate Bone Cement

**DOI:** 10.3390/biomimetics8020262

**Published:** 2023-06-15

**Authors:** Jingyu Liu, Yifan Wang, Yanqin Liang, Shengli Zhu, Hui Jiang, Shuilin Wu, Xiang Ge, Zhaoyang Li

**Affiliations:** 1 Tianjin Key Laboratory of Composite and Functional Materials, School of Materials Science and Engineering, Tianjin University, Yaguan Road 135#, Tianjin 300072, China; liujingyhaha@163.com (J.L.); 3016207462@tju.edu.cn (Y.W.); yqliang@tju.edu.cn (Y.L.); slzhu@tju.edu.cn (S.Z.); h.jiang@tju.edu.cn (H.J.); slwu@pku.edu.cn (S.W.); 2 Key Laboratory of Mechanism Theory and Equipment Design of Ministry of Education, School of Mechanical Engineering, Tianjin University, Tianjin 300354, China

**Keywords:** calcium sulfate bone cement, platelet-rich plasma, biomaterials

## Abstract

Currently, platelet-rich plasma (PRP) is an attractive additive for bone repair materials. PRP could enhance the osteoconductive and osteoinductive of bone cement, as well as modulate the degradation rate of calcium sulfate hemihydrate (CSH). The focus of this study was to investigate the effect of different PRP ratios (P1: 20 vol%, P2: 40 vol%, and P3: 60 vol%) on the chemical properties and biological activity of bone cement. The injectability and compressive strength of the experimental group were significantly higher than those of the control. On the other hand, the addition of PRP decreased the crystal size of CSH and prolonged the degradation time. More importantly, the cell proliferation of L929 and MC3T3-E1 cells was promoted. Furthermore, qRT-PCR, alizarin red staining, and western blot analyses showed that the expressions of osteocalcin (*OCN*) and Runt-related transcription factor 2 (*Runx2*) genes and *β*-catenin protein were up-regulated, and mineralization of extracellular matrix was enhanced. Overall, this study provided insight into how to improve the biological activity of bone cement through PRP incorporation.

## 1. Introduction

Bone damage and bone defect are common problems in clinical orthopedic repair. Currently, bone repair materials are extensively used for treating mainstream bone defects, and it is essential to study bone repair materials with good biological properties [1]. Therefore, it is urgent to develop an injectable bone replacement material with osteoconductive and osteoinductive activity, which can be injected into bone defects during surgery, as well as be self-curing, degradable, and of a certain strength. Bone cement is a moldable paste composed of powder and liquid phases. It can be freely shaped and adapted to the bone defect, serving as an early support for bone cells, and eventually, hardening into a solid phase [2]. Calcium sulfate (CS) is a widely used artificial bioceramic material, and the CS used for bone repair materials is, generally, calcium sulfate hemihydrate (CSH) [3,4]. However, the rate of CS degradation in vivo is too fast and does not match the rate of new bone formation, while the degradation products of CS are acidic, which is detrimental to cell growth and proliferation [5,6]. There are many additions that can be used to improve the bioactivity of CS, which further stabilized the pH of the surrounding environment and regulated the degradation rate, such as calcium carbonate [7], bioactive glass [8,9], and calcium silicate [10,11]. Hydroxyapatite (HA) and carboxymethyl cellulose (CMC) were added in this study to improve the performance of the bone cement. HA is considered a good candidate for various applications in medical fields [12,13], shows pronounced adsorption and differentiation abilities [14], and can regulate the degradation of CSH due to its slow degradation property [15,16]. Scholars indicated that CMC can improve the clinical properties of CS. The CS-CMC putty was easily shaped and adapted to bone defects. [17]. The good shear thinning property of CMC offers good injectability to HA slurry [18], as well as good potential in inducing osteogenic differentiation of osteoblasts [19].

The ideal synthetic bone substitute needs to be both osteoconductive and osteoinductive. Platelet-rich plasma (PRP) has the potential to enhance the bioactivity of bone cement [20]. PRP is a platelet concentrate extracted from autologous blood and contains various growth factors, which can stimulate the proliferation of osteoblasts and fibroblasts, accelerate the differentiation of mesenchymal stem cells (MSC), and promote wound healing in various tissues [21]. Incorporating PRP into brushite cement can accelerate the formation of carbonate apatite [22], and the addition of PRP significantly promotes cell proliferation and differentiation [23]. Past research showed that adding PRP into calcium phosphate bone cement (CPC) carriers enhances the bone regeneration ability of new bone tissue and improves the solubility of CPC. Therefore, PRP is an effective strategy for modulating CPC in bone regeneration in vivo [24,25]. In porous HA/ZrO_2_, PRP can also synergize with heparin sulfate to induce the osteoblast differentiation ability of adipose mesenchymal stem cells [26]. The calcium sulfate precipitation exothermic reaction activates the platelets contained within PRP without the need for agonists, and the activated platelets release multiple biological factors [27]. The opportunely combined CS and PRP (CS-platelet) can induce bone regeneration in critical-sized (non-union) bone defects [28]. Hence, CS-PRP is effective in treating bone defects through stimulating bone regeneration and enhancing the strength of new bone [29]. CSH bone graft combined with autologous platelet gel was more effective than CSH alone in treating maxillary sinus defect. CSH bone graft with PRP showed an increase in bone mineral density (BMD), granulation tissue, and angiogenesis at two weeks after implantation [30].

Although numerous studies demonstrated that PRP admixture into bone graft materials facilitates new bone formation in vivo, the effect of PRP on the properties of injectable bone cement was not elucidated. Considering the controversy over the effectiveness of PRP on bone regeneration, a suitable PRP concentration and incorporation ratio was selected for this work [22]. In the present study, PRP was added into injectable bone cement containing CSH and HA doped with CMC and tested for injectability, washout resistance, compressive strength, setting time, and degradation rate to assess whether it would satisfy clinical requirements. On the other hand, ALP activity tests, alizarin red mineralization assay, *Runx2* and *OCN* gene expression, and *β*-catenin protein expression assays were performed to determine the possible osteogenic effects.

## 2. Materials and Methods

### 2.1. PRP Isolation

PRP was prepared according to the method outlined in [31]. Rabbit whole blood used in the following experiments was purchased from Yishengyuan Biotechnology Co., Ltd. (Tianjin, China), which had a license to keep and use laboratory animals (the license number was SYXK (Tianjin) 2021-0003). The obtained whole blood was centrifuged in an 8 mL PRP preparation tube. A centrifuge machine (TG16-WS, Xiangyi, Hunan, China) was used to centrifuge the tubes at 3500 rpm (1259× *g*) for 10 min for the first step. Secondly, the erythrocytes were precipitated at the bottom of the tube, and the upper plasma portion was then transferred to another tube. Thirdly, the remaining plasma (consisting of platelets and leukocytes) was centrifuged at 4000 rpm (1644× *g*) for 15 min, and the lower one-third portion as PRP was transferred to a new tube.

Platelets (PLTs) were calculated based on the whole blood and PRP content detected via a microscope (Olympus CX41, Olympus Corporation, Tokyo, Japan) and using a hemocytometer after dilution with ammonium oxalate (Phygene Biotechnology Co., Ltd., Fuzhou, China). To ensure that the product released from PRP retained its biological activity, the prepared PRP was applied immediately.

### 2.2. Materials

HA, CSH and sodium carboxymethylcellulose were bought from Mingde Biological Co., Ltd. (Shandong, China). The saline solution was purchased from Siyao Co., Ltd. (Shijiazhuang, China). All laboratory chemical reagents were bought from Aladdin Biochemical Technology Co., Ltd. (Shanghai, China) unless otherwise noted. Isopropanol and anhydrous ethanol were bought from Fengchuan Chemical Reagent Co., Ltd. (Tianjin, China). Phosphate buffered saline (PBS), BCA Protein Assay kit, 4, 6-diamidino-2-phenylindole (DAPI), fluorescein isothiocyanate (FITC), 4% fixative solution, methyl thiazolyl tetrazolium (MTT) assay, Alizarin Red solution (1%, pH 4.2), Western ECL Substrate, and DEPC Water were purchased from Solarbio Technology Co., Ltd. (Beijing, China). Modified–Simulated Body Fluid (SBF) was purchased from Phygene (Fuzhou, China). Cell culture media and 0.05% trypsin-EDTA were purchased from Biosharp Biological Technology Co., Ltd. (Shanghai, China). Fetal bovine serum and double antibody (penicillin and streptomycin) were obtained from Gibco (Carlsbad, CA, USA). The alkaline phosphatase (ALP) assay kit (AKP Microplate test kit) was purchased from Nanjing JianCheng Bioengineering Institute (Nanjing, China). Denaturing buffer (TRIZOL) was acquired from ThermoFisher Scientific (Waltham, MA, USA). Oligo (dT) Primer (3806), Rnase Inhibitor (2313A), dNTP Mixture (4019), M-MLV RTase (2641A), and M-MLV 5x Buffer (SD4041) were purchased from Takara Bio (Beijing, China). Ultra SYBR mixture (low rox) (CW2601H) was purchased from ComWin Biotech Co., Ltd. (Beijing, China). The nitrocellulose membrane was purchased from Millipore (Billerica, MA, USA). RIPA lysis buffer was purchased from Shanghai Yuanye Bio-Technology Co., Ltd. (Shanghai, China). Primary antibody dilution, secondary antibody dilution, SDS-PAGE gels, blocking buffer, and TBST were obtained from Yishengyuan Biotechnology Co., Ltd., (Tianjin, China). The *β*-tubulin (TA-10) was purchased from Zhongshan-Golden Bridge Biotechnology Co., Ltd. (Beijing, China). The *β*-Catenin (bs-1165R) was purchased from Biosynthesis Biotechnology Co., Ltd. (Beijing, China).

### 2.3. Samples Preparation

HA (Ca_10_(PO_4_)_6_(OH)_2_), calcium sulfate hemihydrate (CaSO_4_·0.5H_2_O), and sodium carboxymethylcellulose ([C_6_H_7_O_2_(OH)_2_OCH_2_COONa]_n_) were mixed homogeneously via grinding to create experimental powders. To prepare the specimens for this test, the powder and liquid phases (containing saline and PRP) were mixed at a powder-to-liquid ratio of 1 g:0.9 mL (Table 1), and the cement was placed in cylindrical Teflon molds with a diameter of 6 ± 0.01 mm and a height of 12 ± 0.01 mm until solidification. The preparation process was carried out at 23 ± 1 °C.

### 2.4. Washout Resistance Testing and Injectability Testing

Anti-washout assessment was carried out according to the previous experimental method [32]. The powder (2 g) and liquid (1.8 mL) were mixed into a paste. A 5 mL syringe was used to inject the mixture into 25 mL SBF solution with a constant temperature shaker (SHZ-82, Guohua, Changzhou, China) at 37 °C and 120 rpm. After shaking for 15 min and 30 min, the samples were photographed to observe whether the appearance of the bone cement samples remained intact and determine the degree of turbidity of the SBF solution.

Injectability testing was carried out according to the test method mentioned in [33]. After mixing powder (2 g) and liquid (1.8 mL) into a paste, it was quickly transferred into a 5 mL medical syringe and placed in an injectable test device. The injectability of the mixture was tested using a universal testing machine (WDW-20, Changchunkexin, Changchun, China). The test loading rate was 15 mm/min, and the maximum load was 100 N. The study method was repeated three times for each study group.

### 2.5. Setting Time

The initial and final setting times of different types of cement were determined in a few minutes using the Gillmore needle according to ASTM-C266-15 [22,32]. After mixing, the setting time was measured as the paste hardening time. This test was repeated three times for each study group.

### 2.6. Mechanical Strength Testing

A universal testing machine was used to assess the compressive strength of specimens. The specimens were placed in a constant temperature incubator (DNP-9052BS-Ⅲ, Shanghaixinmiao, Shanghai, China) at 37 °C for 24 h to set. Next, the specimens were set into SBF solution with a constant temperature shaker at 37 °C and 120 rpm for 0, 7, 14, 21, and 28 days. On the respective days, the specimens were removed, dried, and polished. According to ISO 9917-1-2007, the specimens underwent compressive load testing at a crosshead speed of 1 mm/min to test the compressive strength. Each sample was measured in quintuplicate.

### 2.7. Characterization of Bone Cement

An X-ray diffraction (XRD, DX-2700BH, Haoyuan, Dandong, China) instrument was used to identify the material phase. The unsoaked samples P0, P1, P2, and P3 (Table 1), as well as the P0 and P3 samples soaked for 7, 14, 21, and 28 days, were selected and ground to powder for the test. The metal target was Cu (λ = 1.540598 Å). The diffraction angles ranged from 10° to 60°, and the step size was 0.02°.

Field-emission scanning electron microscopy (FE-SEM) and energy dispersive spectroscopy (EDS) (Zeiss Sigma 300, Oberkochen, Germany) were used to analyze the morphology and composition of the sample surface. The samples for FE-SEM observation were sputtered with gold to improve the electrical conductivity.

A simultaneous thermal analyzer (STA 449f3, Netzsch, Selb, Germany) was used to evaluate the thermal stability of the composite bone cement. Dry powders of the unsoaked and soaked groups were tested in an argon atmosphere from room temperature to 800 °C, with a heating rate of 10 °C/min.

Fourier transform infrared spectroscopy (Nicolet IS10, ThermoFisher Scientific, Waltham, MA, USA) was used for the functional groups of the composite bone cement.

The composition and chemical states of different samples were analyzed via X-ray photoelectron spectroscopy (AXIS SUPRA, Kratos, UK). The C1s peak at 284.6 eV was used as the standard for binding energy calibration.

### 2.8. Surface Zeta Potential and Protein Adsorption of Bone Cement

The surface zeta potential of the sample powders was determined using a nano potential analyzer (Zeta-sizer Nano ZS90, Malvern, UK). The samples were ground into powder and sieved (38–45 μm). The test was performed by dispersing 2 mg of CPC powder in 10 mL of PBS solution and sonicating for 5 min. Each group was subjected to this test three times.

Bovine serum albumin (BSA) was dissolved in PBS solution to obtain PBS solutions with different protein concentrations, where the protein concentrations of BSA were 100, 250, and 500 μg/mL, and the sample powder was dispersed in a BSA protein solution at a ratio of 20 mg/mL. The samples were then shaken in the shaker for 3 h and centrifuged (10,000 rpm, 20 min), and the supernatant was extracted. Using the BCA Protein Assay kit, the absorbance of the supernatant at 562 nm was measured via a microplate reader (Synergy H1, BioTek, Burlington, VT, USA), and the protein concentration after adsorption was calculated according to the protein standard curve.

### 2.9. pH of Bone Cement

A precision pH meter (PB-10, Sartorius, Gottingen, Germany) was used to test the pH change in the SBF in which the bone cement samples were immersed. Bone cement samples (Φ 6 × 12 mm) were immersed in 5 mL SBF solution in a centrifuge tube and placed in a thermostatic shaker at 37 °C and 120 rpm. The pH of the solutions in which the bone cement samples were immersed was measured after 1 h, 2 h, 4 h, 8 h, 12 h, 24 h, and 48 h via the pH meter.

### 2.10. In Vitro Degradation Analysis

The prepared samples (Φ 6 × 12 mm) were stored at 37 °C for 48 h. In vitro degradation of the samples was evaluated by immersing them in 10 mL SBF solution for 7, 14, 21, and 28 days in a thermostatic shaker (37 °C, 120 rpm) and refreshing the solution every two days. After stipulated immersion times, the samples were removed, washed with deionized water, dried, and weighed. In vitro degradation was measured using the Formula (1). D is the rate of degradation, and W0 and Wt are the dry weights of the initial and degraded samples, respectively. Each sample was measured in quintuplicate.

D = [(W_0_ − W_t_)/W_0_] × 100% (1)

### 2.11. Hemolytic Rate Measurement

Rabbit blood was centrifuged at 1000 rpm, washed five times with PBS solution to obtain pure red blood cells, and, finally, re-dispersed with 20 mL PBS solution. The bone cement sample (Φ 6 × 1.5 mm) was added to the centrifuge tube, followed by 1 mL of red blood cell suspension. Next, 10% Triton X-100 and PBS solution were used as positive and negative controls, respectively. The centrifuge tube was incubated in a constant temperature incubator at 37 °C for 4 h. After centrifugation, the supernatant was added to the 96-well plate, and the OD value was measured via a microplate Reader at 451 nm. The relative hemolytic rate (RHR%) was calculated using the following formula:

RHR (%) = (OD_Sample_ − OD_PBS_)/(OD_Triton X-100_ − OD_PBS_) (2)

### 2.12. In Vitro Cytotoxicity

The viability of cells was assessed via MTT-based cytotoxicity assay. This experiment was performed following the ISO-10993-5-2009 protocol. For the preparation of extracts, the bone cement sample (Φ 6 × 1.5 mm) was UV-sterilized for 1 h. According to ISO-10993-12-2021, 1.2 g of the bone cement sample was placed in a centrifuge tube and 6 mL of cell culture medium (89% (*v*/*v*) α-minimum essential medium (α-MEM) or Dulbecco’s modified Eagle’s medium (DMEM), and 10% (*v*/*v*) fetal bovine serum (FBS), and 1% (*v*/*v*) double antibody) was poured into each well. After 1 d and 3 d of soaking, the culture solution was used for subsequent cell experiments. The osteoprogenitor cells of MC3T3-E1 and L929 were obtained from the cell bank of the Chinese Academy of Sciences, with all cells collected within ten generations. Specifically, 10^4^ cells/well were seeded in a 96-well plate. After incubating for 24 h, the culture medium was removed and replaced by the extract. After 1 d and 3 d of incubation, the cells were treated with an MTT assay. Briefly, the cells were incubated with MTT solution in a constant temperature incubator for 4 h. Next, the MTT solution was discarded, and an equal volume of dimethyl sulfoxide (DMSO) was added. After being fully dissolved, OD data were measured via a microplate reader at 490 nm. The cell survival rate was calculated using Formula (3).

Cell viability (%) = OD_extract_/OD_control_ × 100% (3)

### 2.13. ALP Assays and Alizarin Red Staining (ARS)

The MC3T3-E1 cells (10^4^ cells/well) were seeded on each specimen (Φ 6 × 1.5 mm) in a 96-well plate. The cells were incubated for 7 and 14 days in a growth medium, which was refreshed every 2 days. After stipulated culture times, cells were lysed with 0.1% Triton X-100 at 37 °C for 1 h. Cell lysates were then used to determine ALP content with an ALP assay kit and total protein with a BCA protein assay kit. The OD value of the former was measured at 520 nm, and the OD value of the latter was measured at 562 nm on a microplate reader. The ALP activity was normalized to the protein amount. Each sample was measured in quintuplicate.

The MC3T3-E1 cells (10^5^ cells/well) were seeded in a six-well plate. After incubation for 48 h in their growth medium, the medium was changed to extract soaked with osteogenic differentiation medium. The cells were incubated for 14 days and 21 days in extract, which was refreshed every 2 days. Firstly, the extracts were discarded and cleaned three times with PBS. In the next step, the cells were fixed with 4% paraformaldehyde for 15 min at room temperature, after which they were discarded, and the cells were washed with PBS three times. Finally, the samples were immersed in ARS staining (pH 7.4) solution for 10 min. A digital camera (EOS80D(W), Canon, Tokyo, Japan) was used to take pictures of the dried samples. For further quantitative analysis, 10% cetylpyridinium chloride (10% *w/v* 10 mM sodium phosphate (pH 7.0)) was added at room temperature for 1 h. The results were measured at 562 nm using a microplate reader (Synergy H1, BioTek, USA).

### 2.14. qRT-PCR Assay

The MC3T3-E1 cells (10^6^ cells/well) were seeded into each well in a six-well plate. The cells were incubated in a culture medium for 48 h, and the culture medium was then changed to an osteogenic differentiation medium. After culturing in the osteogenic differentiation media for 7 and 14 days, cell lysis was performed via a denaturing buffer (TRIZOL), followed by RNA extraction with chloroform. Next, cDNA was obtained via reverse-transcription of RNA extracts at a concentration of 0.1 μg/μL using PrimeScript RT Master Mix (Takara, Otsu, Japan). The target genes included osteocalcin (*OCN*) and Runt-related transcription factor 2 (*Runx2*). Finally, qRT-PCR was performed through an Ultra SYBR mixture (low rox) using the CFX ConnectTM Real-Time System (QuantStudio 1, ThermoFisher Scientific, Waltham, MA, USA). Ct values of target genes were normalized by the *β*-actin to obtain the ΔCt values. The experiments were analyzed using the 2^−ΔΔCt^ method. The primer sequences of the MC3T3-E1 cells are shown in Table 2.

### 2.15. Western Blot Assay

The MC3T3-E1 cells (10^5^ cells/well) were added to a 12-well plate. After 24 h of culture in the growth medium, the growth medium was converted to an osteogenic differentiation medium and cultured for periods of 7 and 14 days. Firstly, cells were harvested and lysed with a RIPA lysis buffer on ice for 20 min. Secondly, the cell lysate was centrifuged at 12,000 rpm at 4 °C for 20 min. Thirdly, a BCA kit was used to obtain the protein concentration. Fourthly, the protein samples were denatured at 95 °C for 10 min and loaded onto SDS-PAGE gels. Fifthly, the proteins were transferred to protein nitrocellulose membranes, and the membranes were blocked at 37 °C for 1 h in a closure buffer. Sixthly, the membranes were placed into a 1:5000 dilution of the primary antibody with a 1:1000 dilution of *β*-Catenin and a 1:2000 dilution of *β*-tubulin at 4 °C for 12 h. The membranes were then cleaned three times with the TBST. Next, 1:5000 dilution of secondary antibody was added to incubate the membranes at 37 °C for 1 h. Finally, the membrane was washed three times with the TBST for 10 min per time to rinse off the secondary antibody, and the results were assessed via chemiluminescence (Champchemi 610 plus, Beijing Sage Creation Technology Co., Ltd., Beijing, China). Grayscale values were analyzed using Image J software (Version 1.8.0_112, National Institutes of Health, Bethesda, MD, USA).

### 2.16. Statistical Analysis

All the experimental data of different groups were analyzed via one-way variance (ANOVA). All data are shown as mean values ± standard deviation (SD). The sample size was equal to or greater than three, and values of *p* < 0.05 were required for results to be statistically significant. * *p* < 0.05, ** *p* < 0.01.

## 3. Results

### 3.1. Platelet Concentration

The values of the platelet counted from the three rabbits are shown in Table 3. It can be seen that the PRP platelet concentration was four times greater than that of whole blood in all three experimental groups; thus, it is a better concentration for promoting bone regeneration [34].

### 3.2. Washout Resistance Testing and Injectability Testing

**Figure 1:**
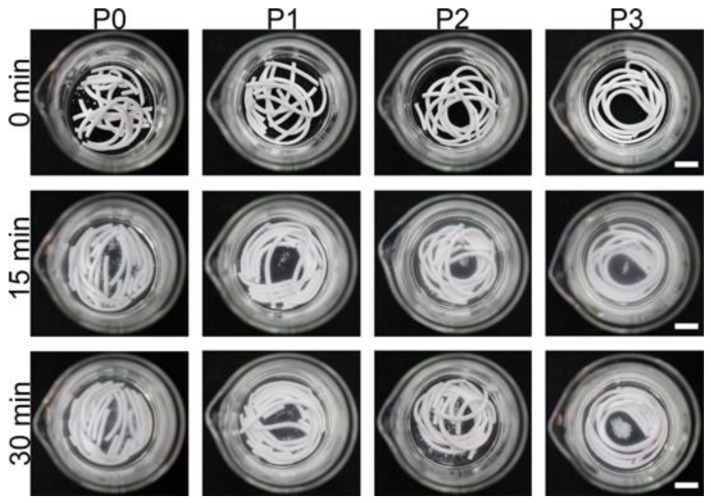
Anti-washout resistance in SBF of different cement groups. Scale bar is 10 mm.

By assessing the anti-washout resistance of the different samples, it can be found that there is no obvious disintegration of paste in all four groups (Figure 1). As shown in Table 4, all four groups had good injectability and did not have solid–liquid separation. Due to PRP increasing the viscosity of the bone cement and acting as a lubricant, the bone cement without PRP addition was injected intermittently, while the addition of PRP could make the injection process occur more smoothly.

### 3.3. Setting Time and Compressive Strength

As shown in Table 5, P0 had the shortest setting time of all samples. With the increasing content of PRP, the setting time increased significantly. The reaction of CSH with water has three time periods: the induction period, the acceleratory period, and the period involving a very slow reaction, i.e., the completion of hydration. The addition of PRP prolonged the hydration process, thus affecting the crystal growth. Although the addition of PRP makes the setting time longer, it is not a problem in practical use in bone surgery. Moreover, its viscosity prevents any disaggregation upon the action of body fluids [35].

The compressive strength of each group of samples without immersion and the compressive strength of each group of samples after immersion are shown in Table 6. The addition of PRP can significantly improve the compressive strength of bone cement. With the increase in immersion time, the mass loss of the sample increased, and the internal space structure became loose; thus, the compressive strength became worse. Although the compressive strength of each group decreased with increasing immersion time, the compressive strength of P3 remains the greatest of all groups at the corresponding time.

### 3.4. Phase and Microstructural Analysis

**Figure 2:**
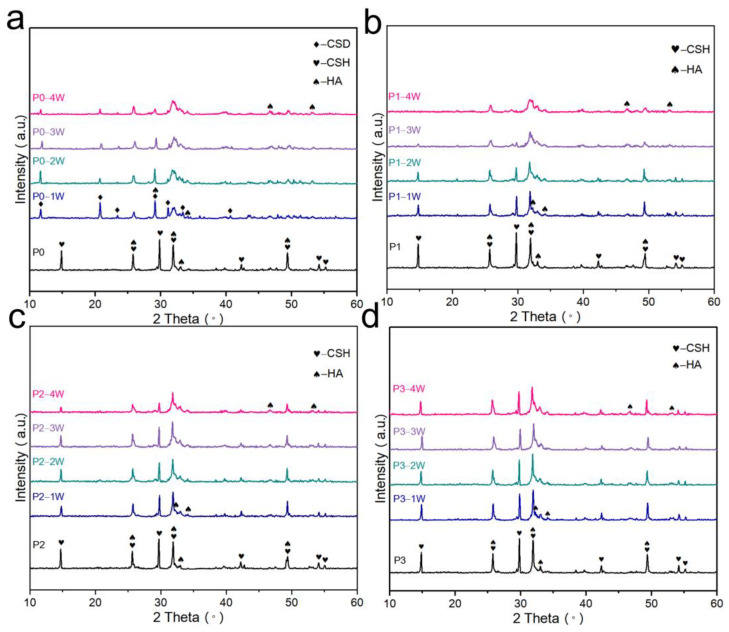
XRD analysis of bone cement powder: (**a**) P0 cement; (**b**) P1 cement; (**c**) P2 cement; (**d**) P3 cement; immersion in SBF for 0, 1, 2, 3, and 4 weeks.

Figure 2 shows XRD patterns of P0 (Figure 2a), P1 (Figure 2b), P2 (Figure 2c), and P3 (Figure 2d) after 0, 1, 2, 3, and 4 weeks of immersion. P0 generated CSD after immersion, while the other three groups did not have characteristic peaks of CSD. Since the detection limit of XRD was about 5%, it can only mean that the other three groups of bone cement did not generate CSD in large quantities. The XRD analysis of bone cement after immersion shows that the intensity of the characteristic peaks decreased with the increase in the immersion time, especially the XRD diffractograms of P0 and P1, thus indicating that the CSH or CSD content decreased.

**Figure 3:**
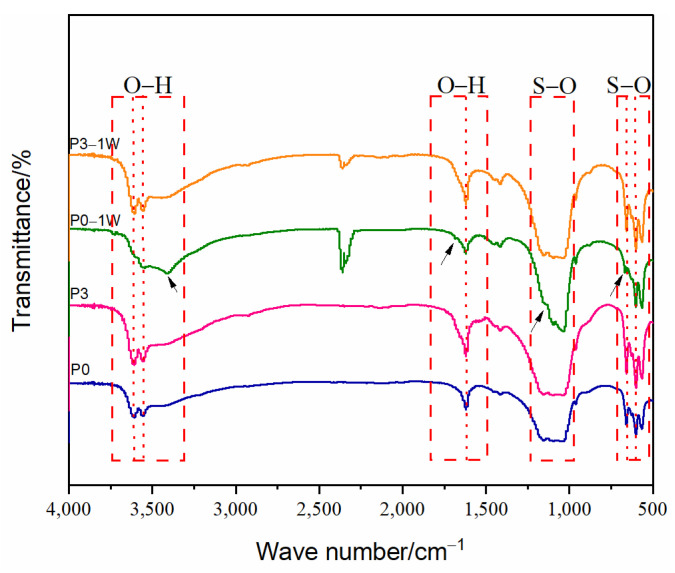
FTIR spectra of different bone cement powders.

Figure 3 showed the infrared spectra of the four groups of bone cement. The infrared spectra of the P0, P3, and P3-1W (P3 sample immersed for 1 week) samples showed CSH characteristic absorption peaks at 3600 cm^−1^, 3550 cm^−1^, 1620 cm^−1^, 650 cm^−1^, and 580 cm^−1^. The infrared spectra of the P0-1W (P0 sample immersed for 1 week) samples showed characteristic absorption peaks of CSD at 3400 cm^−1^, 1680 cm^−1^, 1120 cm^−1^, and 670 cm^−1^ [36]. In agreement with the XRD results, CSD was generated for the P0-1W sample. The characteristic absorption peak of CSD was not seen for the P3-1W sample. This result indicates that the degradation process of P3 did not generate CSD in large quantities.

**Figure 4:**
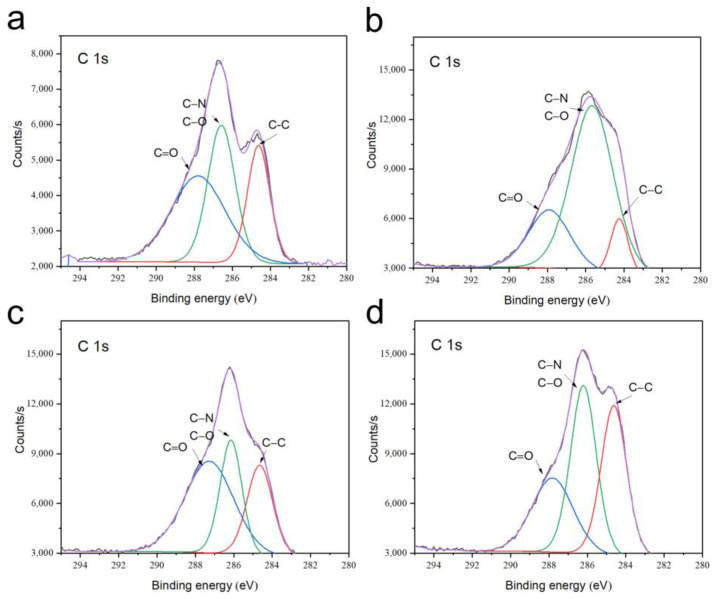
C 1s XPS spectra of P0 (**a**), P3 (**b**), P0-1W (**c**), and P3-1W samples (**d**). The black line is the original curve, and the purple line is the fitting curve.

Figure 4 showed the obtained C 1s XPS spectra of the P0, P0-1W, P3, and P3-1W samples. The C 1s spectra of different samples presented three characteristic peaks. The strongest peak was present at around 286 eV for P3 samples, and the peak was sourced from the C-N in the PRP-containing protein.

**Figure 5:**
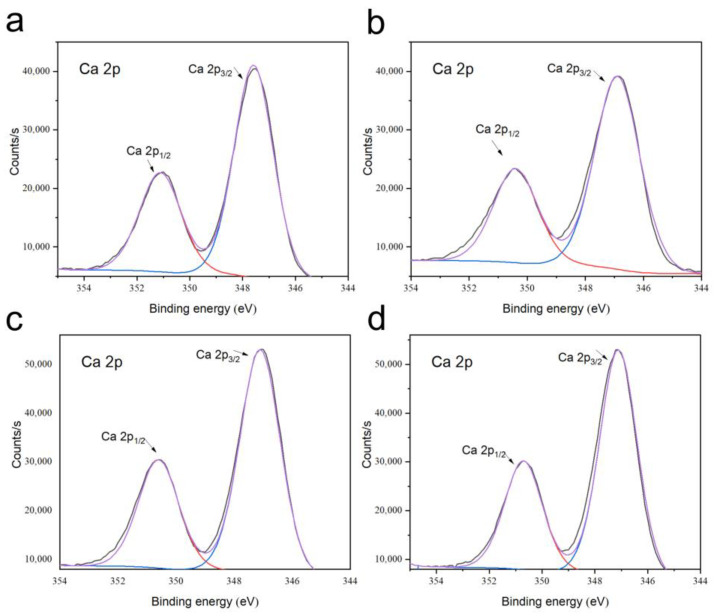
Ca 2p XPS spectra of P0 (**a**), P3 (**b**), P0-1W (**c**), and P3-1W samples (**d**). The black line is the original curve, and the purple line is the fitting curve.

Figure 5 shows the Ca 2p XPS spectra of the P0, P0-1W, P3, and P3-1W samples. The Ca 2p spectra of different samples showed two characteristic peaks. The two peaks were not significantly different between the four groups of 350.7 ± 0.2 eV and 347.1 ± 0.3 eV. Thus, we repeated the assay of thermogravimetry.

**Figure 6:**
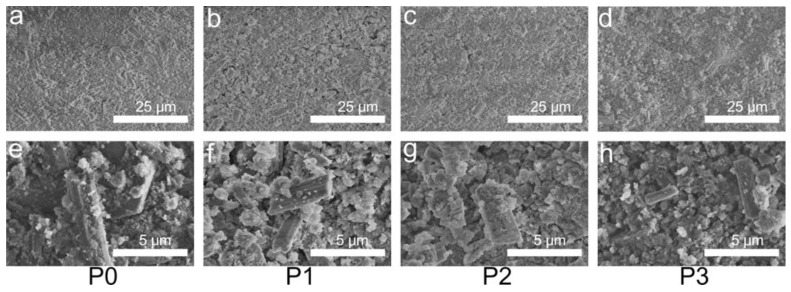
SEM images of P0 (**a**,**e**), P1 (**b**,**f**), P2 (**c**,**g**), and P3 (**d**,**h**). Images of (**a**–**d**) were obtained at 2000×, and images of (**e**–**h**) were obtained at 10,000×.

Figure 6 shows the morphologies of the fracture surfaces of the unsoaked samples. The microstructures of the unsoaked P0, P1, P2, and P3 samples were all agglomerate and columnar. Figure 6 shows that the holes on the sample surface decrease with the increasing content of PRP. In the CSH matrix, the agglomerate phase is HA, and the columnar phase is CSH. Some HA powders were also adsorbed on the upper surface of the CSH. The columnar phase becomes slimmer and shorter with increasing the PRP. The columnar phase of P3 is the smallest.

**Figure 7:**
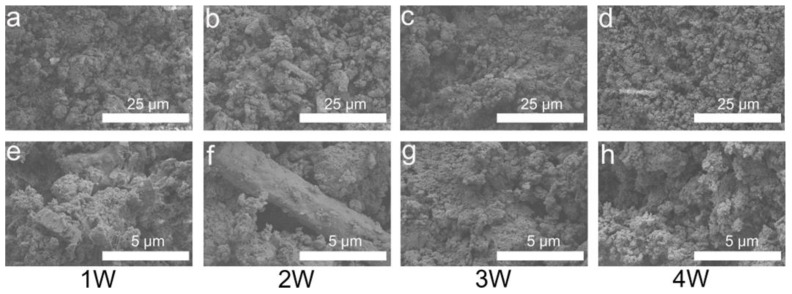
SEM images of P0 after immersion in SBF for 1 (**a**,**e**), 2 (**b**,**f**), 3 (**c**,**g**), and 4 weeks (**d**,**h**). Images of (**a**–**d**) were obtained at 2000×, and images of (**e**–**h**) were obtained at 10,000×.

**Figure 8:**
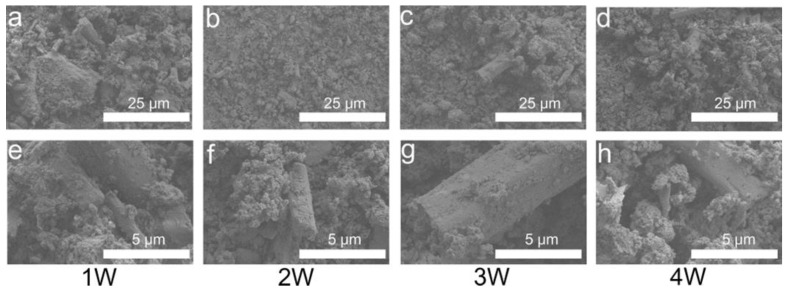
SEM images of P3 after immersion in SBF for 1 (**a**,**e**), 2 (**b**,**f**), 3 (**c**,**g**), and 4 weeks (**d**,**h**). Images of (**a**–**d**) were obtained at 2000×, and images of (**e**–**h**) were obtained at 10,000×.

Figure 7 indicates that the P0 samples generated plate- and rod-like CSD after immersion for one week in SBF solution. CSD was surrounded by agglomerated HA. After immersion for two weeks, rod-like CSD and similar block-shaped CSD were retained. Moreover, the CSD disappeared after immersion for three-to-four weeks, leaving only agglomerated HA. Figure 8 shows that after one week of immersion of the P3 sample, not only was the columnar CSH size different, but the large unhydrated aggregates were also present. The size of the columnar phase present on the surface was unchanged after two weeks of immersion. After three weeks of immersion, only the larger CSH was not degraded. After four weeks of immersion, the CSH debris were the main material present on the surface after degradation. The columnar CSH surrounded by HA was still present.

### 3.5. Thermal Stability

**Figure 9:**
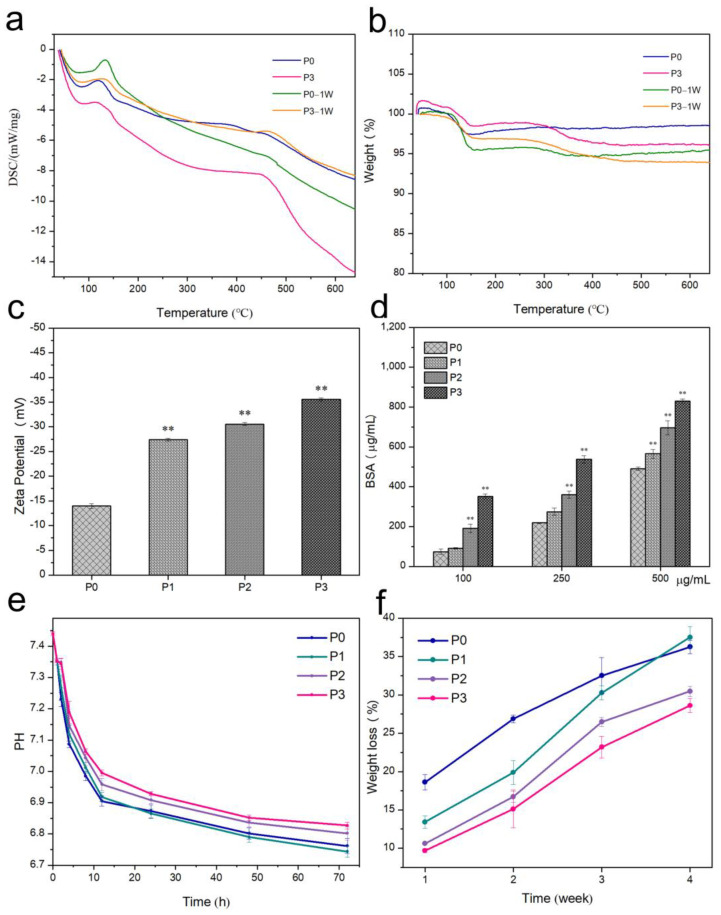
(**a**) DSC curves of 1-week soaked and unsoaked P0 and P3 samples; (**b**) TG curves of 1-week soaked and unsoaked P0 and P3 samples; (**c**) zeta potential of bone cement with different proportions of PRP; (**d**) BSA of bone cement with different proportions of PRP. Error bar indicates mean ± standard deviation: ** *p* < 0.01. *p* values of P1, P2, and P3 groups were obtained via comparison with P0 group; (**e**) pH of bone cement with different proportions of PRP of samples at different time points; (**f**) in vitro degradation behavior was measured using the weight loss ratios of the P0, P1, P2, and P3 samples after immersion in SBF solution for 1, 2, 3, and 4 weeks.

In general, HA is thermally stable below 800 °C, while calcium sulfate dihydrate will lose its water of crystallization at that temperature. Figure 9a showed that P0 and P3 samples had heat absorption peaks at around 120 °C, and P0-1W and P3-1W samples show heat absorption peaks at 130 °C. There was another absorption peak at 450 °C for P3 and P3-1W samples. It was clear that the surface adsorbed and bound water were removed from the samples between 100 and 160 °C. The theoretical crystal water content of CSD is 20.9 wt%, while that of CSH is 6.20 wt% [37]; the CSH content in this paper was 49%, and the thermogravimetric loss was 3.04%. The thermogravimetric losses of P0 and P3 in the figure are 2.53% and 1.51%, respectively, indicating that both P0 and P3 components only contained CSH. The thermogravimetric loss of P3-1W was 2.97%. Assuming that the mass loss in the degradation process was represented by CSH, which should be 2.70%, and all CSD, which should be 9.10%, it was calculated that P3-1W had 95.78% CSH and would generate a small amount of CSD. Due to the addition of PRP, which contained various growth factors and proteins in the P3 sample, thermal decomposition occurred at 300–400 °C due to the presence of amino acids.

### 3.6. Surface Zeta Potential and Protein Adsorption of Bone Cement

As shown in Figure 9c, the potentials of P0, P1, P2, and P3 were −13.95 ± 0.48 mV, −27.40 ± 0.27 mV, −30.55 ± 0.33 mV, and −35.54 ± 0.32 mV, respectively. The zeta potential of P3 is the most negative.

BSA is an acidic protein with an isoelectric point of 4.7. The BSA molecule is negatively charged when in a PBS solution at pH 7.4, and the surface of the sample is also negatively charged, as shown in Figure 9d. Hence, there must be an electrostatic repulsion between the sample surface and the BSA in the solution. Thus, the BSA adsorbed on the P0 powder is less, and most of it remains in the supernatant. The BCA protein assay kit measured the total protein concentration. The PRP contains protein, which will also react with BCA. Therefore, P1, P2, and P3 samples show higher protein content compared to P0. BCA not only reacts with the protein in the solution but also reacts with the protein released from the powder; thus, the value will be larger.

### 3.7. pH of Bone Cement

Figure 9e shows the curves of pH reduction for different groups during immersion. The primary pH of SBF was 7.44 ± 0.01. The pH of the samples decreased rapidly for the first 12 h, and the decreasing trend later became significantly slower. As the immersion time increased, the pH experienced an ever-reducing rate of decrease. P3 had the smallest decrease in pH among the four groups at each time point, reaching 6.83 ± 0.01 at the 72nd hour. The decreasing values of pH for P0, P1, P2, and P3 were 0.68, 0.7, 0.64, and 0.61.

### 3.8. In Vitro Degradation

As shown in Figure 9f, P0 degraded faster in the first two weeks, and the degradation rate decreased significantly in the second two weeks. Moreover, P1, P2, and P3 degraded faster in the second two weeks than in the first two weeks. It can be seen from Figure 7 that P0 generates CSD and then degrades in the first two weeks, while only HA, which degrades at a slower rate, remains in the second two weeks; thus, the degradation rate becomes slower. With the addition of PRP, CSD is no longer generated in large quantities, and in the first week, smaller CSH degrade and larger CSH decompose and become smaller. The rate of degradation was accelerated in the second week because the degradation in the first week broke most of the CSH into smaller pieces that were easier to degrade. In the third and fourth weeks, only a small amount of remaining large-size CSH and CSH incompletely surrounded by HA was present to be degraded, resulting in slower degradation. The main degradation was still from CSH and CSD, while HA was difficult to degrade during the first four weeks.

### 3.9. Hemolytic Rate and In Vitro Biocompatibility

**Figure 10:**
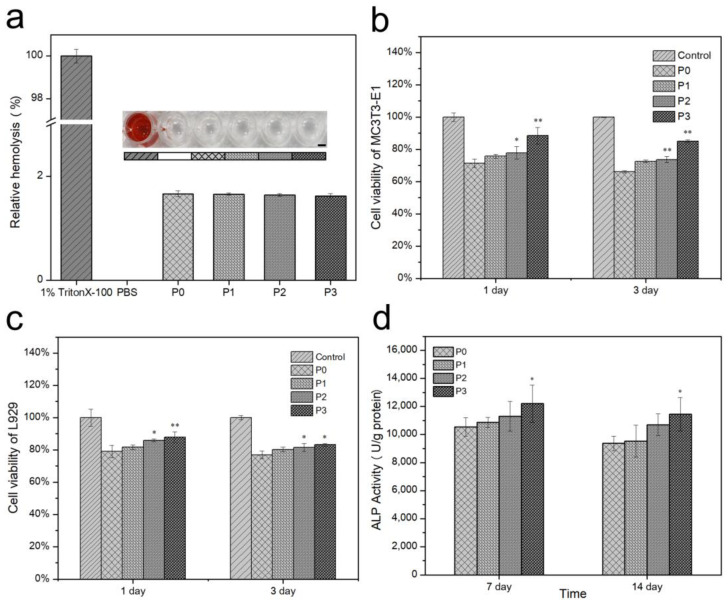
(**a**) Hemolysis rates towards red blood cells: insert figures are optical images of blood supernatant, scale bar was 2 mm; (**b**) MC3T3-E1 and (**c**) L929 viability of bone cement with different proportions of PRP at 1 day and 3 days; (**d**) ALP activity of MC3T3-E1 after culturing for 7 and 14 days normalized to total protein concentration. Error bar indicates mean ± standard deviation: * *p* < 0.05, ** *p* < 0.01. *p* values of P1, P2, and P3 groups were obtained via comparison with P0 group.

It can be inferred from Figure 10a that the hemolytic rates of P0, P1, P2, and P3 were lower than the national standard (5%), being 1.67 ± 0.06%, 1.66 ± 0.02%, 1.65 ± 0.03%, and 1.63 ± 0.04%, respectively. These results indicate that the material has good blood hemocompatibility.

The MTT assay was performed to investigate cell cytotoxic activity. As shown in Figure 10b,c, the P1, P2, and P3 groups showed greater L929 and MC3T3-E1 viability than P0 on days 1 and 3. It can be seen that the addition of PRP promotes cell proliferation. The cellular activity of P0 was only 79.06 ± 3.81%/71.43 ± 2.64% (L929/MC3T3-E1) after one day of culture due to the decrease in pH. It is clear from the figure that pH has a greater effect on the proliferation of MC3T3-E1 cells, and the addition of PRP had a more significant effect on the promotion of cell proliferation. The cell survival rate was significantly lower for 3 days of culture than for 1 day of culture. This result is due to the addition of extracts causing cells to die, resulting in poor cell proliferation activity and, thus, a slower rate of proliferation. Eventually, as the number of days in culture increased, surviving cells became further reduced in number.

### 3.10. ALP and Mineralization

The ALP is an indicator that is commonly used to evaluate the early differentiation activity of osteoblasts. Figure 10d shows the ALP activities of P0, P1, P2, and P3 incubated with MC3T3-E1 after 7 and 14 days. The ALP activities decreased over time. This result is because the level of expression decreases at the onset of mineralization [38]. However, it can be seen that the addition of PRP is favorable for MC3T3-E1 osteogenic differentiation.

To further discuss the effect of PRP addition on osteogenic activity, cell mineralization assays were performed on MC3T3-E1 for 14 and 21 days. ARS staining was used to evaluate the extracellular microenvironment (ECM) mineralization activity. ARS-stained calcium-rich deposits were secreted by cells into a red color [39]. Mineralized nodules were commonly used to assess the osseointegration of MC3T3-E1 cells. In a comparison between the ARS staining photos at 14 and 21 days (Figure 10a), it can be seen that the 21-day sample shows more red mineral granules, indicating an increase in mineral synthesis with time. Comparing the photos of mineral synthesis with and without the addition of PRP samples, it was found that the addition of PRP significantly increased mineral synthesis. Data from the quantitative osteogenesis assay and the ARS staining photos of mineral synthesis show the same results. At each time point, the PRP group had significantly higher mineral concentrations than the control. P3 had the highest ECM mineralization values.

### 3.11. Gene Expression

**Figure 11:**
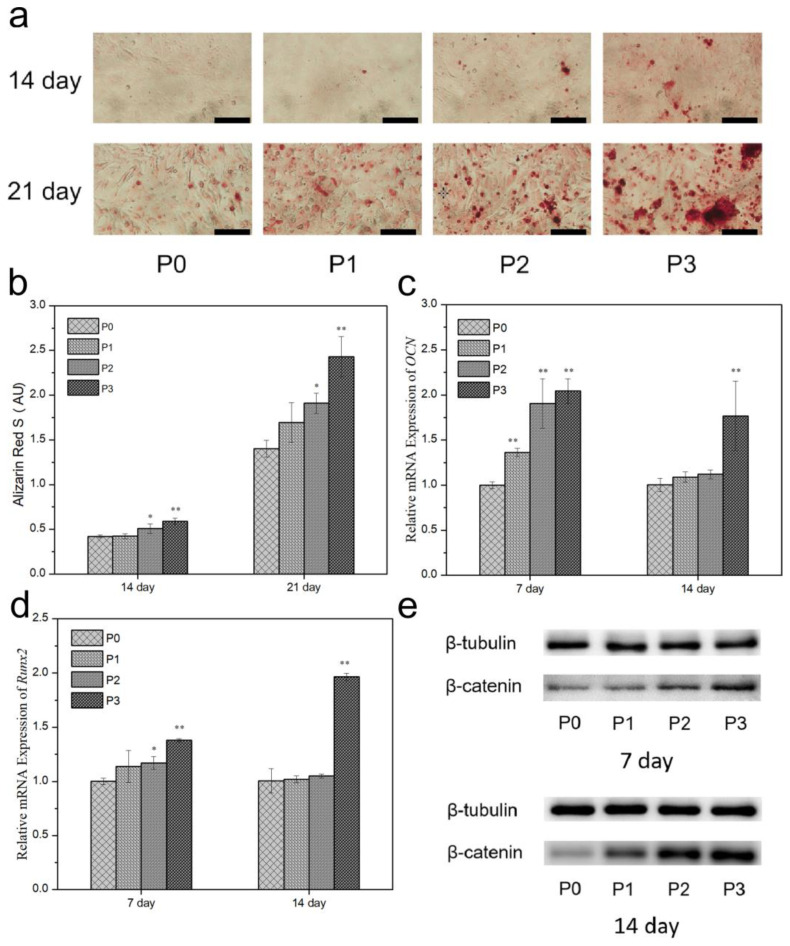
(**a**) ARS staining and (**b**) quantitative analysis of MC3T3-E1 after culturing for 14 and 21 days, with scale bar set at 200 μm; qRT-PCR analysis of expression of (**c**) *OCN* genes and (**d**) *Runx2* genes for MC3T3-E1 after culturing for 7 days and 14 days normalized to *β*-actin expression of cells on P0 on that day. Error bar indicates mean ± standard deviation: * *p* < 0.05, ** *p* < 0.01. *p* values of P1, P2, and P3 groups were obtained via comparison with P0 group. (**e**) Protein expression of *β*-catenin via western blot measured from the MC3T3-E1 cultured for 7 days and 14 days.

Gene expression levels were measured via qRT-PCR. The osteogenic differentiation of MC3T3-E1 was investigated by assessing the expression of the osteogenic genes *Runx2* and *OCN* (Figure 11c,d). P3 samples showed better performance of the two genes than P0 samples after 7 and 14 days of culture. It was demonstrated that the expression levels of both genes were up-regulated due to the addition of PRP. After 7 days of MC3T3-E1 culture, the expression levels of *Runx2* were up-regulated by 1.14, 1.17, and 1.38 times in P1, P2, and P3, respectively; and 1.02, 1.05, and 1.97 times in MC3T3-E1 after 14 days of culture, respectively, compared to P0 samples. The expression of *OCN* in MC3T3-E1 after 7 days of culture was up-regulated about 1.36, 1.91, and 2.04 times in P1, P2, and P3, respectively, compared to P0; and 1.09, 1.12, and 1.77 times in MC3T3-E1 after 14 days of culture, respectively.

### 3.12. Protein Expression

*β*-catenin mainly promotes osteoblast maturation through the Wnt/*β*-catenin pathway, and its loss of function will directly affect the proliferation, differentiation, and maturation of osteoblasts [40]. As shown in Figure 11e, compared to sample P0, the *β*-catenin expression of MC3T3-E1 on P1, P2, and P3 are up-regulated, indicating that the addition of PRP can activate Wnt/*β*-catenin signaling. The protein expression results are consistent with the ALP activity results, and sample P3 has the best performance.

## 4. Discussion

The current study appraised the physical, mechanical, and biological properties generated when PRP was added to CSH/HA bone cement containing CMC. PRP could reduce particle interaction, increase the viscosity of the mixing liquid, and improve the injection performance of bone cement. The addition of PRP was also shown to improve the performance of CPC and increase its viscosity [22]. Due to the lubricating effect of organic matter (PRP) and the viscosity increment of the samples, the bone cement without PRP addition could only be injected intermittently, while the addition of PRP allowed continuous injection of the bone cement and slightly improved injectability.

However, the washout resistance properties of the experimental groups were not significantly different from those of the control. The solidification of bone cement is the result of dissolution and precipitation processes. Studies showed that the admixture of a drug in the solid or liquid phase affects the setting reaction of bone cement, resulting in reduced porosity, smaller precipitated crystals, and other physicochemical and mechanical properties [41].

The reaction of CSH with water has three time periods: the induction period, the acceleratory period, and the period involving a very slow reaction, i.e., the completion of hydration. The induction period starts immediately after the CSH powder is mixed with the aqueous solution. The CSH dissolves, and the solution becomes supersaturated concerning calcium and sulfate ions, leading to the precipitation of CSD crystals [42]. CMC was added to this experiment: this polymer absorbs water, and lower water volumes do not allow uniform and complete dissolution of the CSH [43]. CMC was found to be particularly effective as a CSD growth inhibitor and elongated the induction period [44]. The presence of CMC in this study led to a competitive chemical process taking place, which led to the blockage of the setting reaction. Moreover, the presence of PRP and HA [45] affected the hydration of CSH and prolonged the setting time. The addition of PRP as a liquid phase with reduced water content also affected crystal growth.

It could be seen from the SEM images that the addition of PRP made the CSH crystals finer, with fewer pores and a compact microstructure. It was shown that PRP contains proteins and growth factors that have a good affinity for calcium cement, and the edges and pores of bone cement particles are blocked by PRP gel-like contents [22]. Several studies previously confirmed that fibrin-based biomaterials increase the mechanical properties of scaffolds [46]. One of the key specific considerations for bone cement is its biomechanical properties [47]. Fibrin in PRP may form a network structure that prevents ion dissolution and water molecules from gaining entry, and PRP adsorbed on the solid surface hinders crystallization.

As mentioned above, it can be seen that with the addition of PRP, the sample crystal structure was made into a finer, more compact combination of CSH and HA, and the porosity was smaller, thus increasing the compressive strength. The ideal bone substitute material should have controlled biodegradability, create space for new bone tissue, and, eventually, be replaced by mature bone tissue [48]. In the case of rapid CS degradation, the addition of CMC and PRP not only prevents the hydration of CSH but also slows down its degradation rate.

Only trace amounts of CMC contained in the control specimen cannot prevent the hydration of CSH into CSD in the SBF; thus, P0 became CSD and HA after a week of immersion. Adding PRP prevented its hydration, the concentration of dissolved ions was not yet sufficient to generate CSD, and the immersion fluid was renewed. Thus, CSD was not generated in large quantities.

A moderate degradation speed during the first 4 weeks is essential to prolong implantation in the human body and avoid erupting dissolution and poor structural stability. The degradation rate of P3 is the lowest in each week, and in the fourth week, the degradation rate of P3 decreases to 28.62 ± 0.93%. Compared to the degradation rate of P0 of 36.26 ± 0.86%, the result indicates that the addition of PRP made the structure more stable and had a positive effect in terms of prolonging the implantation time.

It can be seen from Figure 9e that after sample immersion, the solution shows an alkaline to acidic pH shift, which is caused by the generation of acidic products in CS degradation [49]. The addition of PRP decreased the degradation rate and slowed down the acidification process, which affected the rate of pH decrease. Although the increased concentration of calcium ions and the altered pH value formed locally after CS degradation may play a stimulating role in osteoblast differentiation [50], rapid degradation and an acidic environment are prone to in vivo inflammation, which is also detrimental to bone healing. An improved pH environment can prevent inflammatory responses in vivo.

The platelet activation secretes a variety of growth factors, such as VEGF, TGF-*β*, IGF, and PDGF, which bind to receptors on the cell membranes of stromal cells and fibroblasts, promoting the proliferation and differentiation of these repair cells at the fracture end and facilitating vascular regeneration and granulation tissue formation. The acceleration of bone healing depends on the proliferation of osteoblasts, on the one hand, and the local osteogenic activity of new bone cells, on the other hand. MTT test, ALP activity assay, and calcium nodule staining of the samples showed that the cell proliferation capacity, secretion of ALP, and formation of calcium salts became stronger after the effect of PRP. Thus, PRP has a stimulating effect on osteoblast proliferation, on the one hand, and enhances its osteogenic activity, on the other hand, which can strengthen the repairing effect at the site of bone healing.

The addition of PRP decreased pH and reduced the effects of acidosis on cellular function. Acidosis reduces cell proliferation and suppresses mineralization and cellular alkaline phosphatase activity [51]. In osteoblasts, *Runx2* promotes early differentiation and suppresses late differentiation. *OCN* is a marker gene for late osteogenic differentiation and is required for calcium salt deposition and bone matrix formation. However, *Runx2* can promote the transcription and expression of *OCN* [52]. It is also shown that TGF-*β* can promote the transcriptional activity of *Runx2* [53].

*Runx2* and *OCN* are important landmarks in osteoblast differentiation, and Wnt/*β*-catenin signaling is an essential signaling pathway that regulates bone metabolism. In this study, the mRNA expression and the protein expression in P3 were higher than that in P0 at both time points, confirming that the addition of PRP had a clear osteogenic-inducing activity. Several past studies showed that the addition of PRP in human clinical trials increases BMD in maxillary sinus defects, reduces defect volume, and increases granulation, osteoblast, and angiogenesis [30].

## 5. Conclusions

This study shows that PRP incorporation into bone cement improves the injectability and mechanical properties of the bone cement. Despite the increase in setting time, it produced finer crystal morphologies, which had a positive effect on both subsequent degradation and pH drop. Moreover, the addition of PRP significantly improved bioactivity. The addition of PRP also accelerated cell proliferation and enhanced osteogenic differentiation. This new PRP-incorporated type of bone cement optimized the internal structure of bone cement and significantly improved bioactivity. Therefore, the PRP alternative to liquid-phase admixed bone cement has good prospects for application in bone defect repair. However, animal experiments were not performed, and the mechanism of its alleviation of degradation needs further study. In the future, we will implant the material into the body, and study whether it has a positive effect on bone regeneration and tissue regeneration in vivo.

## Figures and Tables

**Table 1:** Composition of different groups.

Cement Type	Solid Phase Composition (g/g)	Liquid Phase Composition (Normal Saline/PRP [mL/mL])	Final Liquid/Solid Ratio (mL/g)
P0	HA (49%) ^a^+ CSH (49%) ^b^ + CMC (2%) ^c^	5:0	0.9
P1	HA (49%) ^a^+ CSH (49%) ^b^ + CMC (2%) ^c^	4:1	0.9
P2	HA (49%) ^a^+ CSH (49%) ^b^ + CMC (2%) ^c^	3:2	0.9
P3	HA (49%) ^a^+ CSH (49%) ^b^ + CMC (2%) ^c^	2:3	0.9

^a^ HA, hydroxyapatite; ^b^ CSH, calcium sulfate hemihydrate; ^c^ CMC, sodium carboxymethylcellulose.

**Table 2:** Primer sequences in qRT-PCR gene expression analysis.

Gene	5′-3′	Primers
*OCN*	forward	GAACCAAGAAGGCACAGACAGA
	reverse	GGCGGGACACCTACTCTCAT
*Runx2*	forward	AGCAGGAGGGCAATAAGGTAGT
	reverse	TCGTCACAAGCAGGGTTAAGC

**Table 3:** Platelets number in whole blood and PRP of three rabbits.

Group	PLTs Count in Whole Blood (10^9^/L)	PLTs Count in PRP (10^9^/L)
1	343.33 ± 25.42	1508.33 ± 35.31
2	381.00 ± 13.14	1534.00 ± 33.95
3	330.00 ± 16.39	1466.00 ± 42.06

**Table 4:** Injectability of bone cement with different proportions of PRP.

Cement Type	Injectability (%)
P0	96.51 ± 0.24
P1	96.73 ± 0.12
P2	96.95 ± 0.11 *
P3	97.01 ± 0.07 *

The data are presented as mean ± standard deviation: * *p* < 0.05. *p* values of P1, P2, and P3 groups were obtained via comparison with P0 group.

**Table 5:** Initial and final setting times of bone cement with different proportions of PRP.

Cement Type	Initial Setting Time (min)	Final Setting Time (min)
P0	22.78 ± 0.76	89.25 ± 1.31
P1	29.78 ± 0.41 **	120.06 ± 1.38 **
P2	34.46 ± 0.52 **	122.57 ± 0.51 **
P3	37.85 ± 1.11 **	133.23 ± 0.21 **

Data are presented as mean ± standard deviation: ** *p* < 0.01. *p* values of P1, P2, and P3 groups were obtained via comparison with P0 group.

**Table 6:** Compressive strength of bone cement with different proportions of PRP after immersion in SBF for 0, 1, 2, 3, and 4 weeks.

Cement Type	Compressive Strength (MPa)				
	0 Week	1 Week	2 Weeks	3 Weeks	4 Weeks
P0	5.06 ± 0.43	1.99 ± 0.34	0.91 ± 0.14	0.79 ± 0.06	0.76 ± 0.04
P1	5.96 ± 0.26 **	2.28 ± 0.19	1.70 ± 0.33 **	1.19 ± 0.21	0.88 ± 0.15
P2	6.61 ± 0.22 **	3.59 ± 0.16 **	2.59 ± 0.35 **	2.20 ± 0.30 *	1.65 ± 0.12 **
P3	6.84 ± 0.31 **	3.72 ± 0.26 **	2.74 ± 0.37 **	2.74 ± 0.37 **	1.81 ± 0.28 *

Data are presented as mean ± standard deviation: * *p* < 0.05, ** *p* < 0.01. *p* values of P1, P2, and P3 groups were obtained via comparison with P0 group.

## Data Availability

Not applicable.
